# Novel Nanocomposites for Luminescent Thermometry with Two Different Modalities

**DOI:** 10.3390/molecules29061350

**Published:** 2024-03-18

**Authors:** Masfer Alkahtani, Yahya A. Alzahrani, Abdulaziz Alromaeh, Philip Hemmer

**Affiliations:** 1 Future Energy Technologies Institute, King Abdulaziz City for Science and Technology (KACST), Riyadh 11442, Saudi Arabia; yalzhrani@kacst.edu.sa; 2 Microelectronics and Semiconductors Institute, King Abdulaziz City for Science and Technology, P.O. Box 6086, Riyadh 11442, Saudi Arabia; eng.abdulaziz2@gmail.com; 3 Institute for Quantum Science and Engineering, Texas A&M University, College Station, TX 77843, USA; prhemmer@exchange.tamu.edu; 4 Department of Electrical and Computer Engineering, Texas A&M University, College Station, TX 77843, USA

**Keywords:** nanocomposites, upconversion nanoparticles, fluorescent nanodiamonds, silicon vacancy

## Abstract

In this work, we successfully integrated fluorescent nanodiamonds (FNDs) and lanthanide ion-doped upconversion nanoparticles (UCNPs) in a nanocomposite structure for simultaneous optical temperature sensing. The effective integration of FND and UCNP shells was confirmed by employing high-resolution TEM imaging, X-ray diffraction, and dual-excitation optical spectroscopy. Furthermore, the synthesized ND@UCNP nanocomposites were tested by making simultaneous optical temperature measurements, and the detected temperatures showed excellent agreement within their sensitivity limit. The simultaneous measurement of temperature using two different modalities having different sensing physics but with the same composite nanoparticles inside is expected to greatly improve the confidence of nanoscale temperature measurements. This should resolve some of the controversy surrounding nanoscale temperature measurements in biological applications.

## 1. Introduction

The field of luminescent nanothermometry has emerged as a vital and innovative area of research, particularly for its capability to accurately detect temperature fluctuations at a nanoscale. This technique utilizes a variety of advanced materials, including organic dyes [1], highly fluorescent semiconducting polymer dots [2,3], diamond color centers [4], silicon carbides [5,6], hexagonal boron nitrite [7], fluorescent silica particles [8], upconverting nanoparticles [9,10], gold nanoparticles, and carbon dots [11]. These materials are key in implementing luminescent nanothermometry, each offering unique properties that contribute to precise temperature measurements.

Despite its advancements, luminescent nanothermometry has faced challenges, particularly in the context of cellular temperature measurements. Recent debates in the scientific community have centered around the accuracy of temperature readings obtained in various experiments [12]. Critics argue that the reported temperature changes are theoretically implausible based on fundamental thermodynamic principles, suggesting the presence of unknown cellular interferents that may lead to erroneous readings. To circumvent this issue, a multi-modal temperature measurement approach has been proposed [4]. This method involves using multiple temperature probes, each based on different physical mechanisms, to simultaneously measure temperature. By doing so, the impact of any potential interferents can be minimized, enhancing the reliability of the readings.

Toward this goal, efforts have been put together to engineer hybrid nanocomposites containing different nanoparticles for nanothermometry [13], drug delivery [14,15], and other biological applications. In view of the prior works, the different fluorescent nanoparticles were introduced to the hybrid nanocomposites by either functionalization or direct mixing. This makes them susceptible to unwanted separation mechanisms, especially at different pH levels in living cells. Therefore, a single-particle version of these composites is needed. Ideal candidates should exhibit features such as excitation and detection within the biological transparency window, high-temperature sensitivity, ultra-small size for high spatial resolution, a high molar absorption coefficient at the excitation wavelength coupled with a high fluorescence quantum yield, stability against blinking and photo-bleaching for real-time imaging, robust surface chemistry, biocompatibility, and finally non-toxicity [4].

In the field of optical temperature sensing, fluorescent lanthanide-doped upconversion nanoparticles (UCNPs) have shown exceptional promise [16,17]. These materials share critical optical, chemical, photochemical, and photophysical properties, making them ideal fluorescent markers. UCNPs doped with lanthanide ions such as Er, Tm, and Ho have demonstrated their efficacy in optical temperature sensing within living cells, showcasing a high-temperature resolution. Their mechanism of temperature sensing is based on the relative thermal populations of excited states, which affects the fluorescence intensity ratio (FIR) of two transitions. This FIR method is an improvement over single-transition fluorescence measurements, as it is less susceptible to signal losses and fluctuations in excitation intensity, which could otherwise compromise temperature measurement accuracy [9,17,18,19].

In addition, fluorescent nanodiamonds (FNDs) with negatively charged nitrogen-vacancy (NV) centers have been successfully employed to measure local temperatures in living human cells with remarkable precision and spatial resolution. The NV center leverages a magnetic spin transition sensitive to thermal lattice expansion, providing a highly accurate temperature reading. Finally, FNDs can also make use of optical transitions, such as the zero-phonon line of the silicon-vacancy (SiV) center [16,20,21]. 

In this study, we present a straightforward yet effective methodology for synthesizing a composite particle consisting of both fluorescent nanodiamonds (FNDs) containing silicon vacancies and upconversion nanoparticles based on rare-earth-doped sodium yttrium fluoride (NaYF_4_:Yb,Er). FNDs with silicon vacancies were carefully chosen to be the core of the synthesized FND@UCNP composition due to their narrow and distinguished emission peak shifted from the UCNP emission peaks. It worth mentioning that the FND were cleaned with molten potassium nitrate reported in [22,23] to enhance their optical properties. To verify the successful incorporation of nanodiamonds within the host UCNP crystals, we employed a combination of advanced characterization techniques such as X-ray diffraction (XRD), electron microscope tomography, and optical characterizations. Furthermore, we performed optical temperature sensing within the biologically relevant temperature range of 298 to 310 K. The reported results demonstrated a simultaneous correlation in temperature readings from the two nanothermometers in the synthesized nanocomposites, underlining their effectiveness and reliability in precise temperature measurement. This innovative approach not only ensures the integration of these two distinct types of nanoparticles but also enhances their collective properties, making them suitable for a range of advanced applications.

## 2. Results and Discussion

**Figure 1:**
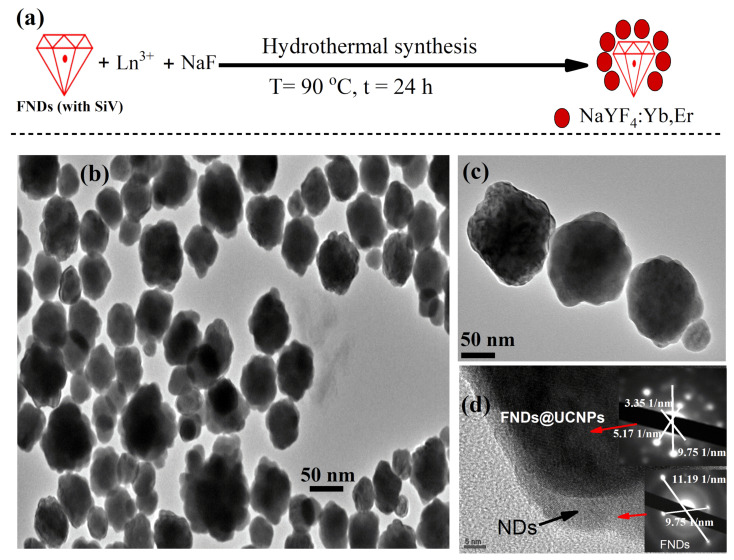
(**a**) Schematic representation of the hydrothermal synthesis process for fluorescent nanodiamond (FND) and upconversion nanoparticle (UCNP) shells, conducted in a hydrothermal vessel with a Teflon liner at 90 °C for 24 h. (**b**) Low-magnification transmission electron microscopy (TEM) image showcasing the formation of ND@UCNP shells, revealing well-crystallized and evenly dispersed nanoparticles. (**c**) High-magnification TEM image of the ND@UCNP shells, highlighting detailed structural features. (**d**) Enhanced TEM close-up of the FNDs@UCNPs, illustrating the distinct overgrowth of the UCNPs shell on top of the FNDs, where darker particles signify UCNPs and lighter, less distinct particles indicate the presence of FNDs. ((**d**), inset) TEM diffraction pattern taken from FNDs and UCNPs shell which confirm the presence of the FNDs inside the grown UCNPs shells.

Composite particles consisting of fluorescent nanodiamonds (FNDs) and sodium fluoride-based upconversion nanoparticles (NaYF_4_:Yb,Er) in a core–shell structure are illustrated in Figure 1a. This synthesis of the studied nanocomposites is detailed in Section 3. High- and low-magnification transmission electron microscopy (TEM) images of these particles are shown in Figure 1b,c. The TEM images show a well-crystallized and well-dispersed nature of the FND@UCNP nanocrystals in a core–shell structure with an average size of 50 nm. Figure 1d shows clear evidence of the UCNPs shell grown over the FNDs nanocrystals. The dark coating observed corresponds to the shell of the UCNPs, a fact that was subsequently verified by the TEM diffraction pattern, revealing lattice spacings of 3.35 Å (110) and 5.714 Å (100) plus a large diffraction pattern spacing of cubic diamond phase at 2.06 Å (111) confirming the presence of the diamond at the core of FNDs@UCNPs nanocomposites. Similarly, the layer with lower contrast shown in Figure 1d, at the edge of the FNDs@UCNPs nanocomposites, indicates the existence of FNDs at the core, which was also confirmed through TEM diffraction patterns, demonstrating a lattice spacing of cubic diamond phase at 2.06 Å (111).

**Figure 2:**
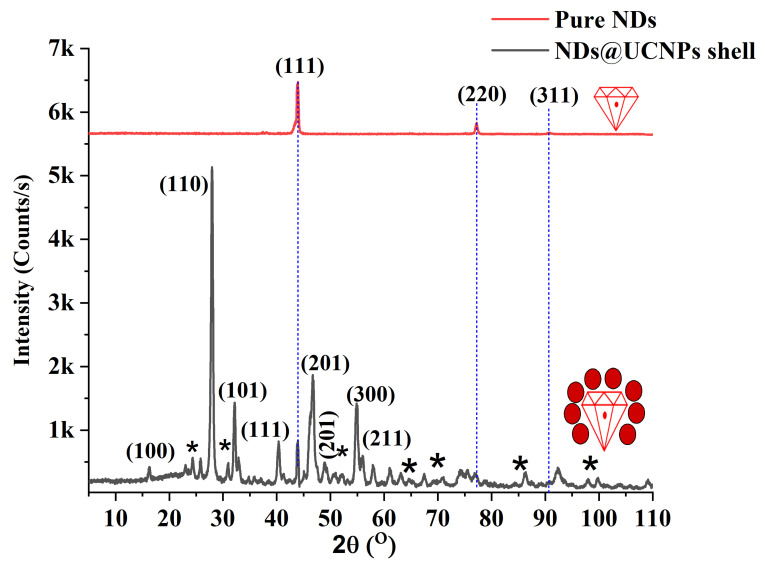
The X-ray diffraction (XRD) pattern of the pristine nanodiamonds (NDs) is presented in red and exhibits distinct peaks at 43°, 76°, and 92°. These peaks correspond to the (111), (220), and (311) crystallographic planes of diamond, indicating the pure crystalline nature of the NDs. In contrast, the XRD pattern of the synthesized ND@upconversion nanoparticle (ND@UCNP) nanocomposite is shown in black. This spectrum demonstrates additional features, suggesting the successful integration of the UCNP shell onto the ND crystals. Notably, the new peaks and shifts in the black curve signify changes in the crystal structure due to the formation of the composite material. The asterisks marked on the ND@UCNP XRD spectrum identify the XRD peaks originating from the quartz substrate.

To compare the structural composition of both pure NDs and NDs coated with upconversion nanoparticles (ND@UCNP nanocomposite), X-ray analysis was employed. In Figure 2, the diffraction peaks of the composition of the ND@UCNP nanocomposite are concurrently observed (depicted by the black spectrum). These diffraction peaks closely align with the characteristic peaks corresponding to the alpha phase of NaYF_4_ (JCPDF: No. 00-028-1192) and NDs (JCPDF: No. 01-089-3441). Moreover, the X-ray diffraction (XRD) pattern of the pristine NDs (illustrated by the red spectrum) reveals distinctive peaks at 43°, 76°, and 92°, corresponding to the (111), (220), and (311) planes of diamond (JCPDF: No. 01-089-3441). The concurrent detection of these peaks in the ND@UCNP composition suggests the successful growth of the UCNP shell over the ND crystals. This observation is consistent with the results obtained from transmission electron microscopy (TEM) images, reinforcing the validity of the UCNP growth on the ND crystals. 

Studying and analyzing the optical properties of the composition of the ND@UCNP nanocomposites is another tool to prove the successful fabrication of a core–shell structure. For this purpose, a laser-scanning confocal microscope was custom-made. The design and construction details of this confocal laser scanning microscope are thoroughly described in Section 3 of our research. The key feature of this microscope is its capability to scan samples with multiple laser excitations at specific wavelengths. Following excitation, the fluorescence emitted by the sample is collected back through the same objective lens. This fluorescence signal is then efficiently routed to the detection unit of the microscope. 

**Figure 3:**
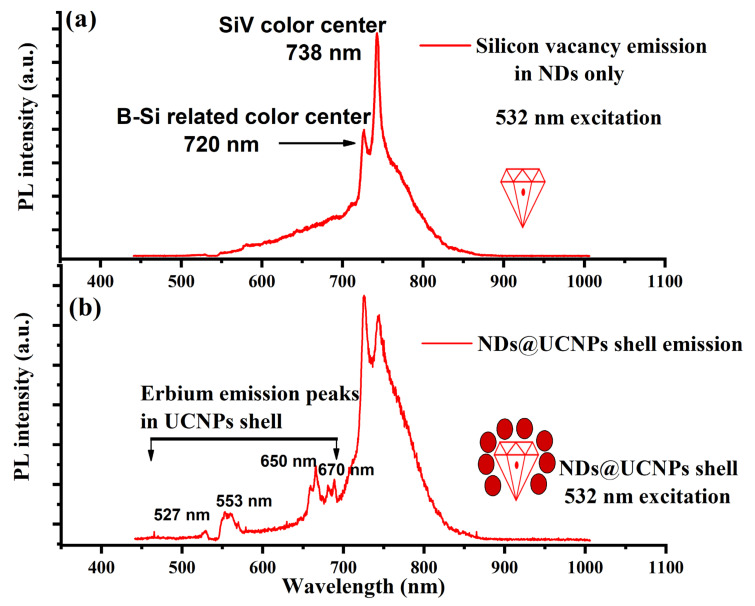
(**a**) This figure illustrates the optical emission characteristics of fluorescent nanodiamonds (FNDs) under green excitation. The observed photoluminescence spectrum distinctly highlights two key emission features: a boron–silicon-related peak at 720 nm and a silicon-vacancy (SiV) color center peak at 738 nm. These specific wavelengths are indicative of the unique electronic and optical properties of the FNDs. (**b**) This panel displays the optical emission spectrum of the newly synthesized ND@UCNP shell composites. The photoluminescence spectrum here reveals a significant overlap between the emission wavelengths of the FND color centers and the characteristic erbium emission peaks. This overlap suggests effective energy transfer mechanisms within the composite material, highlighting the successful integration of the UCNP shell with the FNDs.

For the optical characterization of the composition of ND@UCNP samples, the nanocomposite crystals were uniformly spin-coated onto a quartz coverslip to create a thin layer. This method ensures a homogeneous distribution of the nanoparticles across the surface, which is crucial for consistent and accurate optical measurements. Once prepared, these samples were mounted onto our custom-built confocal microscope for detailed examination. First, the FNDs without the UCNP shell were characterized separately using a 532 nm laser diode at an intensity of 50 W/cm^2^. Figure 3a shows the optical emission with a strong and narrow SiV center emission with a pronounced ZPL peak at 737 nm. In addition to the SiV center peaks in the optical spectrum, we also observed a sharp peak centered at 720 nm which could be attributed to the boron–silicon-related color center reported in previous studies [24]. Next, the ND@UCNP composition sample was characterized with green laser excitation, giving a richer optical emission spectrum as shown in Figure 3b. In this optical spectrum, we observed a clear overlap between the erbium ion emission peaks centered at 527 nm, 553 nm, 650 nm, and 670 nm and the SiV peak from the FNDs in the core of the nanocomposites.

**Figure 4:**
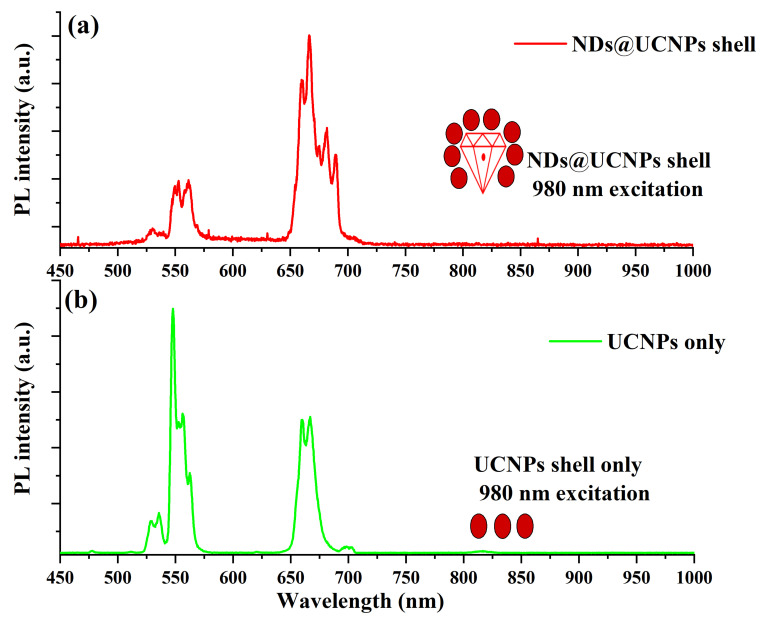
(**a**) This figure illustrates the upconversion (UC) fluorescence spectrum of the ND@UCNP nanocomposite when exposed to a 980 nm laser excitation. The spectrum reveals a notable quenching in the green emission region, which is attributed to the high concentration of P1 centers within the fluorescent nanodiamonds (FNDs) at the core of the ND@UCNP nanocomposite crystals. The P1 centers, indicative of high nitrogen or boron concentrations, play a crucial role in altering the fluorescence characteristics by absorbing and re-emitting light in specific wavelength ranges. This phenomenon underlines the intricate relationship between the composite’s structural composition and its optical properties. (**b**) This panel showcases the typical upconversion emission spectrum of upconversion nanoparticles (UCNPs) under the same 980 nm laser excitation. The emission spectrum of the UCNPs is characterized by distinct peaks, reflecting the unique luminescent properties of these nanoparticles in the FNDs.

Similarly, we excited the ND@UCNP composition sample with a 980 nm laser diode, but at a lower intensity of 20 W/cm^2^. Figure 4a demonstrates the typical upconversion emission of the UCNP shell; however, the green emission peaks at 527 nm and 553 nm, respectively, are strongly quenched compared to the red emission peaks at 650 nm and 670 nm. We propose that this green emission quenching occurred due to the cubic phase in the UCNPs. For comparison, Figure 4b illustrates the upconversion emission of the UCNPs alone under 980 nm laser excitation at the same laser power density. 

The process underlying the upconversion luminescence observed in the ND@UCNP nanocomposites is a fascinating interplay of physics and material science. This luminescence is a result of a series of sequential energy transfers involving two types of ions: the sensitizer (Ytterbium, Yb^3^⁺) and the activator (Erbium, Er^3^⁺). These ions play distinct roles in the upconversion process, each contributing to the unique luminescent properties observed in the UCNPs. The initial stage of this process begins with the Yb^3^⁺ ions, which have a notably large absorption cross-section in the range of 950–1000 nm. When exposed to near-infrared (NIR) light within this range, Yb^3^⁺ ions absorb the first photon, transitioning to their ^2^F_5/2_ excited state. This excited state of Yb^3^⁺ is pivotal as it serves as the energy donor in the subsequent transfer process. The absorbed energy is then transferred from the excited Yb^3^⁺ to the Er^3^⁺ ions, specifically promoting the Er^3^⁺ to the semi-resonant metastable ^4^I_11/2_ level. This level is significant because of its relatively long lifetime, measured in milliseconds, allowing for the accumulation of energy.

The process continues as a second NIR photon, again absorbed by Yb^3+^, further excites the Er^3+^ ions, elevating them to even higher excited states. This sequential photon absorption and energy transfer is a key aspect of the upconversion mechanism. Following these excitation steps, the Er^3+^ ions undergo multiple nonradiative relaxations. As a result of these relaxations, the Er^3+^ ions emit two distinct and sharp emission lines. These lines, centered at 527 nm, 553 nm, and 650 nm, correspond to the transitions from the ^2^H_11/2_, ^4^S_3/2_, and ^4^F_9/2_ excited states of Er^3+^ to its ^4^I_15/2_ ground state [9]. Each of these emission lines represents a different energy transition within the Er^3+^ ions, contributing to the rich luminescent spectrum of the UCNPs. The efficient transfer of energy from Yb^3+^ to Er^3+^, followed by the controlled release of this energy as visible light, is what makes these UCNPs so valuable for applications requiring high-intensity, stable, and tunable luminescence. 

**Figure 5:**
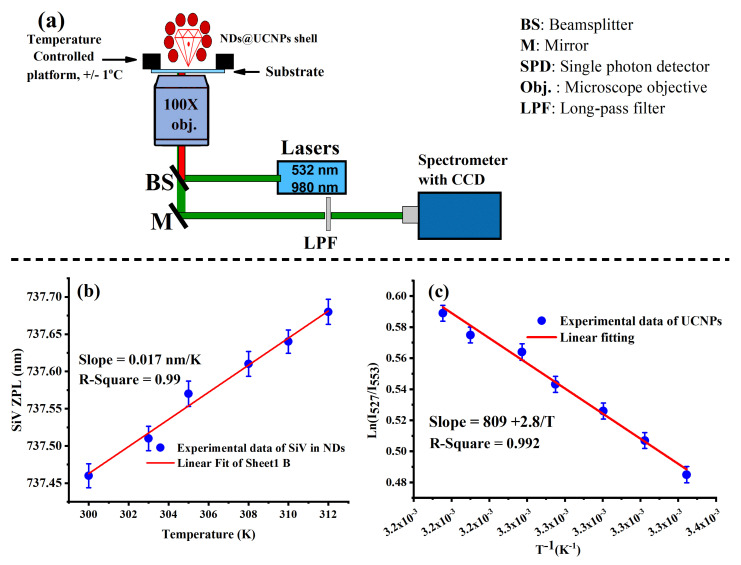
(**a**) An illustration of the optical setup used for temperature sensing. The optical setup is equipped with green and NIR laser light, a temperature-controlled platform, and a spectrometer. (**b**) Shows a linear fitting of the SiV zero-phonon line (ZPL) position, demonstrating its change as a function of temperature over a range of 300 K to 312 K. (**c**) Shows the temperature dependence of the upconversion luminescence emission spectrum of NaYF₄:Yb,Er UCNP shell when excited with a 980 nm laser, recorded over the same temperature range (300 K to 312 K).

After performing structural and optical characterization of the ND@UCNP nanocomposites, we explored their application in optical temperature sensing. For this, optical temperature sensing was performed over a narrow temperature range, specifically between 298 K and 310 K. This range is particularly relevant for biological systems, making it crucial for applications in biotechnology and medical research. To facilitate this study, a solution containing 1 mg/mL of the sample type was prepared. This solution was then uniformly spin-coated onto a quartz substrate, ensuring a well-dispersed layer of nanocrystals. The prepared sample was then mounted onto a compact heater, which was integrated into the laser scanning microscope setup as illustrated in Figure 5a. This heater was crucial for controlled temperature modulation during the optical temperature measurements. Prior to conducting the actual experiments, the heater’s temperature was calibrated over a broader range (298–330 K) to rule out any potential confusion caused by laser-induced heating. 

Subsequent to calibration, the sample was scanned using a green laser (532 nm) to create an optical scan of the ND@UCNP nanocomposites with SiV centers. It was observed from the optical emission spectroscopy results that the zero-phonon line (ZPL) peak of the SiV centers shifts towards the red end of the spectrum as the temperature increases. Figure 5 particularly highlights the ZPL shifts at two representative temperatures, 300 K and 310 K. Such shifts are indicative of the temperature-dependent behavior of the SiV centers and are critical for understanding their role in temperature sensing.

Fitting the experimental data of the optical temperature sensing of SiV color centers in FNDs to a linear equation gives a slope of 0.017 nm/K, as demonstrated in Figure 5b. This slope provides a calibration curve for temperature measurements, enabling us to quantitatively interpret the temperature-induced shifts in the ZPL peaks. These results align well with previous studies on optical thermometry using SiV color centers in diamond nanocrystals, conducted over a similar temperature range. To estimate the SiV sensor sensitivity, with an integration time of 10 s, we were able to detect temperature changes as small as 0.2 K. This level of sensitivity aligns with the reported critical optical temperature sensing threshold for a similar integration time reported in [16,21,25,26]. 

Next, the optical temperature dependence of upconversion (UC) emission in the synthesized ND@UCNP nanocomposites was measured, again within a biocompatible temperature range. To this end, the same sample was subjected to a 980 nm laser scan, and the changes in UC luminescence were measured, which corresponded to variations in temperature. The temperature dependence of UC green emission was recorded over a range from 298 K to 315 K. This experiment was conducted under a low-power 980 nm laser excitation at 20 W/cm^2^. Such a low power setting is critical to prevent any local heating that might otherwise influence the accuracy of optical temperature sensing measurements.

UCNPs are adept at sensing temperature changes. This ability is attributed to the Boltzmann populations in two thermally coupled excited state levels (^2^H_11/2_, ^4^S_3/2_) of the erbium (Er^3+^) ion. The fluorescence intensity ratio (FIR) of transitions from these erbium excited states (^2^H1_1/2_ and ^4^S_3/2_) to the erbium ground state (^4^I_15/2_) serves as a functional measure of temperature changes. This relationship can be mathematically represented by Equation (1), as follows:

(1) $$\mathrm{FIR}=\frac{I_{527}}{I_{552}}={\mathrm{Ce}}^{-\frac{\Delta E}{\mathrm{KT}}}$$

In this equation, C is an empirical constant, encompassing the degeneracies of the erbium Er^3+^ (^2^H_11/2_, ^4^S_3/2_) excited states, emission cross-sections, and the fluorescence frequencies of the two green transitions. k represents the Boltzmann’s constant, T is the temperature, and ΔE signifies the energy separation between the two thermally coupled levels in the Er^3+^ excited states. 

Figure 5c presents an intriguing linear dependence between the FIR and the inverse temperature, based on experimental data. The linear fitting of FIR against the inverse of temperature (1/T) revealed that the energy difference between the thermally coupled excited states is approximately 810 cm^−^¹. Next, we calculated the sensitivity as a function of temperature. Our data revealed that the ND@UCNP nanocomposites exhibited sensitivities in the range of 1.2–1.3% K^−^¹ within the biological temperature window. This performance aligns well with the high relative sensitivities of UCNPs reported in previous studies [9,17].

After obtaining the calibration curves of the two nano-thermometers within the ND@UCNPs nanocomposites, simultaneous measurements of temperature were performed. Upon dual laser excitations with green and NIR, optical spectra from the same spot in the optical scan were acquired concurrently. By setting the heating stage temperature to 298.25 K, the temperature reading by both SiV center and UCNPs in the nanocomposites were found to be 298.35 K and 298.55 K, respectively. The results of these measurements are summarized in Table 1, which details the temperature readings obtained using both nanothermometers simultaneously over the biological temperature range from 298 K to 312 K.

Notably, the small difference observed in the temperature measurements between the two modalities fell within the sensitivity limits of each method. 

## 3. Materials and Methods

### 3.1. Materials

The chemicals used in our experiments, including Ytterbium (III) Chloride Hexahydrate, Yttrium (III) Chloride Hexahydrate, and Erbium (III) Chloride Hexahydrate, are categorized under lanthanide elements, commonly known as rare earth chlorides. Additionally, we utilized lithium hydroxide, ammonium fluoride, oleic Acid, 1-octadecene, methanol, ethanol, cyclohexane, and deionized water in our procedures. All these reagents were sourced from Sigma-Aldrich (St. Louis, MO, USA) and were used as received, without undergoing any further purification processes. The quality and consistency of these chemicals were integral to the success of our experimental outcomes, ensuring reliable and reproducible results in our studies.

### 3.2. FND Cleaning in Molten Potassium Nitrate (KNO_3_)

In this study, FNDs containing SiV color centers with an average size of 45–50 nm were sourced from Adamas Nanotechnologies, based in Raleigh, USA. Prior to the growth of UCNPs on FNDs in a core–shell structure, FNDs underwent a careful cleaning process to remove all graphitic shells on the surface of the FNDs. To achieve this, we employed a well-established molten potassium nitrate (KNO₃) treatment. This process, conducted at a high temperature of 600 °C for 10 min, is recognized for its effectiveness in refining nanodiamond surfaces, as outlined in several References [22,23]. The primary goal of this treatment was not only to clean the FNDs but also to smooth their sharp edges, transforming them into a more rounded form. The cleaning process began by thoroughly mixing 2 mg of FNDs (equivalent to 400 µL from a 5 mg/mL FND suspension in deionized water) with 1 g of KNO₃ powder. This mixture was prepared in an agate mortar to ensure a uniform and homogeneous blend. Once mixed, the resulting compound was placed in a quartz boat, which was then positioned inside a Thermolyne 21,100-tube furnace. Here, the mixture underwent an annealing process at 600 °C for 10 min.

Post-annealing, the sample was allowed to cool to room temperature. It was then dissolved in water and subjected to centrifugation. The resultant pellet was resuspended in 2 mL of water and briefly sonicated for 10 s in an ultrasonic bath. This sonication step is crucial as it helps to disperse any aggregated particles caused by residual KNO_3_, ensuring a more uniform suspension. Following sonication, the sample was centrifuged again. This washing and centrifugation process was repeated five times, a critical step in thoroughly removing all traces of salt residue from the nanodiamonds. 

### 3.3. Hydrothermal Synthesis of FND@UCNP Nanocomposites

Nanodiamond–NaYF composite materials were produced through a modified hydrothermal method [13]. Initially, a solution containing 2 mL of 0.25 M of trivalent ion precursors (comprising 80% Yttrium, 18% Ytterbium, and 2% Erbium) was prepared. This solution was made by dissolving Yttrium, Ytterbium, and Erbium chlorides (all in hexahydrate forms) in the KNO_3_-treated nanodiamond suspension with a concentration of 1 mg/mL. The mixture was then continuously stirred at a speed of 600 rpm for a duration of 48 h. To this stirred solution, 5.0 mL of water and 2.5 mL of a 0.80 M sodium fluoride solution were added. The mixture was stirred again at 600 rpm, this time for 30 min. Following the removal of the stir bar, the mixture was then transferred to a Teflon-lined autoclave. This autoclave was subsequently heated to 90 °C and maintained at this temperature for 24 h in an oven. Post-heating, the autoclave was allowed to cool down to room temperature naturally. The resultant product was then washed three times using ethanol. For the purpose of precipitation, a centrifuge was employed, operating at 6000 rpm for 10 min. The final product was a colorless powder, which was then dispersed in 5 mL of ethanol.

### 3.4. Transmission Electron Microscope Imaging

Transmission electron microscopy (TEM) micrographs of the synthesized ND@UCNP shell nanocomposites were acquired using a high-resolution Titan 200 kV ST (FEI) electron microscope. Before proceeding with the TEM imaging, an important preparatory step was undertaken to optimize the quality of the images. The TEM grids, which serve as the platform for holding the nanoparticle samples, were subjected to a plasma treatment. This treatment is critical for several reasons: firstly, it effectively removes any organic residues, dust particles, and other contaminants from the grids. Such impurities, if left unaddressed, can interfere with the clarity and accuracy of the TEM images. Secondly, the plasma treatment enhances the hydrophilicity of the TEM grids. 

For the TEM imaging process, a few drops of the nanoparticle sample solution were carefully placed onto the prepared TEM grids. Drop casting is a widely used method for depositing samples on TEM grids, as it allows for a controlled and uniform distribution of the nanoparticles across the grid surface. After the drop-casting procedure, the TEM grids with the samples were dried under vacuum. This drying step is crucial as it removes any remaining solvent, leaving behind a thin, evenly distributed layer of nanoparticles on the grid. This preparation ensures that the nanoparticles are in an optimal state for imaging, free from distortions or aggregations that could compromise the quality of the TEM micrographs.

### 3.5. Custom-Made Confocal Laser Scanning Microscope for Optical Characterizations

For the purpose of optically characterizing the synthesized ND@UCNP shell nanocomposites, we designed a confocal laser scanning microscope. This custom-built microscope consists of a 4f imaging system, multiwavelength laser sources, a home-built spectrometer, and a photon counter, which are integral components for detailed spectral analysis. The optical system was powered by two continuous wave (CW) lasers: a 532 nm green laser for FND excitation and a 980 nm laser for UCNP excitation. These lasers were carefully selected for their high stability and Gaussian beam profile, which ensures minimal spectral error and precise illumination of the samples.

To prepare the samples for optical measurements, we used a drop-casting method to deposit the FNDs and FND@UCNP nanocomposites on quartz substrates. This technique provides a uniform and thin layer of nanoparticles, which is essential for consistent optical characterization. The substrates with the nanoparticle samples were then positioned on the x–y–z stage of the confocal microscope. The optical scanning of the samples was performed using an x–y scanner, and the optical emission spectra emitted from the FNDs and FND@UCNP nanocomposites were collected through the same microscope objective. These spectra were then analyzed using our custom-made spectrometer. This spectrometer was outfitted with an 1800-grooves/mm diffraction grating, enabling a fine spectral resolution of 0.03 nm. The combination of a sensitive camera and a single photon counter in the spectrometer setup was crucial for accurately detecting and quantifying the emitted photons.

### 3.6. Optical Temperature Sensing with the Cleaned FNDs and UCNPs

For conducting accurate optical temperature measurements using silicon-vacancy (SiV) color centers in fluorescent nanodiamonds (FNDs) and upconversion nanoparticles (UCNPs), we integrated a highly precise and well-controlled heating stage into our confocal microscope setup. This heating stage was essential for maintaining and adjusting the temperature of the samples during the experiments. The process began with careful focusing on the selected optical spot of the samples using the x–y scanning feature of the confocal microscope. Once the target area was accurately identified and focused upon, the heating stage was activated. This stage methodically heated the FND and UCNP samples across a predetermined temperature range, which is a critical factor for investigating the temperature-dependent fluorescence properties of these materials. As the temperature of the samples increased, the fluorescence emissions from the SiV color center’s zero-phonon line (ZPL) and the green emission from the UCNPs were meticulously recorded. Capturing these emissions is vital as they provide direct insights into the temperature-induced changes in the optical properties of the nanoparticles. The recorded fluorescence emissions were then analyzed using a high-resolution spectrometer attached to the optical confocal microscope. This spectrometer played a crucial role in precisely determining the spectral characteristics of the emissions, allowing for detailed analysis of the fluorescence shifts and intensities.

The final step in the process involved plotting and analyzing the recorded shifts in the ZPL of the SiV centers or the UCNP emissions as a function of the increasing temperature. This analysis was pivotal in understanding the relationship between temperature and the optical properties of these nanomaterials. By carefully examining these shifts, we gained valuable insights into the thermal sensitivity and stability of the FNDs and UCNPs, which are essential for their potential applications in areas such as nanotechnology, biomedicine, and materials science. The integration of a precise heating stage with the advanced optical capabilities of the confocal microscope provided a robust and accurate system for conducting optical temperature measurements at the nanoscale.

## 4. Conclusions

In this study, we have successfully developed and characterized ND@UCNP nanocomposites with a core–shell structure, employing an innovative hydrothermal growth method. Our synthesis process yielded ND@UCNP nanocomposites featuring well-dispersed particles with an average size of approximately 50 nm. 

The successful formation of a UCNP shell over fluorescent nanodiamond (FND) cores was rigorously verified through a series of sophisticated analytical techniques. High-resolution transmission electron microscopy (TEM) imaging provided clear visual evidence of the core–shell architecture, revealing the intimate integration of the UCNPs with the FNDs. X-ray diffraction analysis further confirmed this structural integration, showing distinct patterns indicative of both components within the composite material. Furthermore, the optical properties of the synthesized ND@UCNP nanocomposites were investigated under dual excitation conditions. This analysis revealed unique spectral features, demonstrating the successful integration of the optical characteristics of both NDs and UCNPs within the composite structure.

In summary, the synthesis and application of core–shell ND@UCNP nanocomposites for simultaneous optical temperature sensing were performed. The obtained nanocomposites showed an accurate and simultaneous measurement of temperature using two different modalities having different physics. This makes them highly suitable for biomedical applications, including sensitive temperature sensing, and high-resolution bio-imaging. 

## Figures and Tables

**Table 1:** A direct correlation between temperature measurements using two different modalities.

Heating Stage Temperature (K)	Temperature with SiV in FNDs (K)	Temperature with UCNPs (K)	Temperature Difference between Sensors (K)
298.25	298.35	298.55	0.2
300.35	300.55	300.45	0.1
303.35	303.45	303.65	0.2
305.25	305.35	305.45	0.1
308.25	308.45	308.55	0.1
310.35	310.55	310.35	0.2
312.15	312.35	312.55	0.2

## Data Availability

The data presented in this study are available upon request from the corresponding author.
